# West Nile Virus Infection in Travelers Returning to United Kingdom from South Africa

**DOI:** 10.3201/eid2502.172101

**Published:** 2019-02

**Authors:** Vivak Parkash, Kate Woods, Liana Kafetzopoulou, Jane Osborne, Emma Aarons, Katharine Cartwright

**Affiliations:** Royal Hallamshire Hospital, Sheffield, UK (V. Parkash, K. Cartwright);; Public Health England, Porton Down, UK (K. Woods, L. Kafetzopoulou, J. Osborne, E. Aarons)

**Keywords:** West Nile virus, West Nile fever, flavivirus, rhabdomyolysis, myositis, encephalitis, West Nile neuroinvasive disease, United Kingdom, South Africa, viruses

## Abstract

West Nile virus (WNV) is an arthropod-transmitted flavivirus that causes West Nile fever and may infrequently cause neuroinvasive disease in humans. We present 2 cases of confirmed WNV infection, 1 of severe encephalitis and 1 of mild febrile illness, in a couple returning to the United Kingdom from South Africa.

West Nile virus (WNV) is a mosquitoborne flavivirus maintained in an enzootic cycle between culicine mosquitoes and birds. Approximately 80% of human infections are subclinical, although symptomatic cases can vary from influenza-like symptoms to neurotropic manifestations (*1*). West Nile neuroinvasive disease, which manifests as encephalitis, meningitis, or acute flaccid paralysis, occurs in <1% of all cases (*2*)*.*

To date, human disease caused by WNV has been attributed to WNV phylogenetic lineages 1 and 2 (*3*). Lineage 2 consists of viruses found in South Africa, Madagascar, and, more recently, in Europe and Russia (*4*). Lineage 2 was previously described as less pathogenic than lineage 1, but it has subsequently been demonstrated that lineage 2 can also lead to neuroinvasive disease (*5*). We describe a couple from the United Kingdom with confirmed WNV infection who were admitted to a hospital in February 2017 on return from a holiday to South Africa, where they most likely acquired WNV in the Kruger National Park region.

A previously healthy 76-year-old woman was admitted with acute confusion to Doncaster Royal Infirmary, Doncaster, UK. She and her husband had returned that day following a 3-week holiday visiting family in South Africa (Appendix Figure). The patient and her husband had stayed in Johannesburg, except for a 5-day safari in Kruger National Park 5 days into their trip. They had both sustained several mosquito and tick bites. No malaria prophylaxis had been taken, and no pretravel advice had been sought.

The patient boarded an airplane independently in Johannesburg but her condition deteriorated in flight; on arrival, she was transferred directly to the hospital. She was febrile, hypotensive, and agitated, with a Glasgow Coma Score fluctuating between 7 and 14, and had intermittent vacant episodes (Table). She was empirically treated for meningoencephalitis with intravenous cefotaxime, amoxicillin, and aciclovir. Because of her travel history, intravenous artesunate was added for possible malaria and doxycycline for possible rickettsial infections. On the second day of admission, the patient was transferred to the Department of Infection and Tropical Medicine (Sheffield, UK).

**Table:** Diagnostic investigations for the index case of West Nile virus infection in a woman from the United Kingdom who traveled to South Africa*

Test	Result	Reference range	Day of testing†
Blood tests			
White cell count	12.2 × 10^9^ cells/L	3.5–9.5 × 10^9^/L	1
C-reactive protein	81 mg/L	0–5 mg/L	1
Creatine kinase	593 U/L	25–200 U/L	1
Glucose	7 mmol/L	3–6 mmol/L	1
Liver function tests			
Alkaline phosphatase	60 IU/L	30–130 IU/L	3
Aspartate aminotransferase	33 IU/L	0–32 IU/L	3
Gamma glutamyl transferase	78 IU/L	0–40 IU/L	3
Gamma glutamyl transferase	155 IU/L	0–40 IU/L	11
Alanine aminotransferase	35 IU/L	0–33 IU/L	3
Alanine aminotransferase	37 IU/L	0–33 IU/L	6
Malaria testing			
Rapid antigen detection‡	Negative		1
Thick/thin blood films	Negative		1, 2, 3
Lumbar puncture			
Visual appearances	Clear, colorless CSF		2
Opening pressure	22 cm H_2_O	5–18 cm H_2_O	2
Leukocyte count	102 × 10^6^ cells/L	<5 × 10^6^ cells/L	2
Leukocyte differential	80% polymorphonuclear cells; 20% lymphocytes		
CSF protein	1.24 g/L	0.15–0.45 g/L	2
CSF glucose	3 mmol/L	3.3–4.4 mmol/L	2
CSF molecular assays			
Herpes simplex virus DNA	Not detected		2
Varicella zoster virus DNA	Not detected		2
Enterovirus RNA	Not detected		2
Meningococcus DNA	Not detected		2
Pneumococcus DNA	Not detected		2
Listeria DNA	Not detected		2
Serology and blood cultures			
Blood cultures	No growth after 5 d		1, 3, 5
HIV screen	Negative		3
Syphilis screen	Negative		3
Imaging and EEG			
Chest radiograph	Unremarkable		1
Contrast MRI of the brain	No leptomeningeal enhancement; established right cerebellar infarct		5
EEG	Rhythmic irregular delta slow-wave activity (suggestive of encephalopathy)		11

*CSF, cerebrospinal fluid; EEG, electroencephalogram; MRI, magnetic resonance imaging. †After hospital admission. ‡*Plasmodium* lactate dehydrogenase (pLDH) test (OptiMAL-IT, Bio-Rad, http://www.bio-rad.com).

After discussion with the Imported Fever Service, we sent blood, cerebrospinal fluid (CSF), and urine samples to the Public Health England Rare and Imported Pathogens Laboratory (RIPL) for testing (Appendix Figure). By ELISA, serum was strongly positive for WNV IgM but negative for IgG. WNV RNA was detected in urine by reverse transcription PCR (RT-PCR), confirming the diagnosis. CSF test results were abnormal (Table) but negative for both WNV RNA and IgM. Follow-up serum specimens taken 2 weeks later demonstrated seroconversion of WNV IgG (ELISA). Subsequent whole-genome sequencing of virus detected in the urine sample demonstrated that it belonged to WNV lineage 2.

Empirical antimicrobial drug treatment was stopped, and the patient’s Glasgow Coma Score improved to 14, with residual mild cognitive impairment, ongoing balance disorder, and improving myalgia. Inflammatory markers and all other blood parameters normalized before discharge 5 weeks later. At follow-up 12 weeks after presentation, the patient’s cognition had improved, but she still required use of a cane for persistent balance disturbance.

One day after the index patient’s admission, her 72-year old husband was admitted with an influenza-like illness. He had stage 3 chronic kidney disease but no other concurrent conditions. Urine and serum samples were obtained, and test results confirmed that he, too, had had a recent WNV infection. WNV IgM and IgG were positive in serum 5 days after symptom onset; WNV RT-PCR on urine was negative. He had no neurologic component to his illness and made a rapid clinical recovery.

WNV was initially isolated from a febrile patient in Uganda in 1937 (*6*). The virus was not correlated with severe human disease until the 1990s, when outbreaks in Europe were associated with higher rates of West Nile neuroinvasive disease (*7*)*.* WNV is sporadically reported from South Africa (*8*). Imported cases to the United Kingdom are rare, and autochthonous infections have yet to be reported. One other RT-PCR–confirmed case of acute WNV infection has been reported in a traveler returning to the United Kingdom since 2012; 7 other clinically suspected cases have shown compatible WNV serologic test results (Public Health England, unpub. data).

Clinicians should bear in mind the value of urine sampling to detect WNV RNA by RT-PCR in addition to serologic testing. WNV is excreted in high concentrations in urine and may be detected for several weeks postinfection, whereas detectable virus in serum and CSF is transient and generally not present by the time of symptom onset (*9*)*.* Detection of WNV IgM in CSF is used to confirm neuroinvasive disease. WNV IgG appears shortly after IgM but remains positive in the long term and is therefore not a useful marker of recent infection unless seroconversion is demonstrated.

Clinicians should be alert to the possibility of arthropod-borne infections in travelers returning from South Africa. These include WNV, African tick-bite fever, chikungunya, and *Plasmodium falciparum* malaria. In addition, an outbreak of arboviral infections, particularly Sindbis virus, had been reported in the Johannesburg area coinciding with the travel dates of the patients we report (*10*)*.*

Appendix Figure giving timeline of West Nile virus exposure risk, testing, and progress of illness in woman who returned to the United Kingdom from South Africa.
