# Supplementary material for: West Nile Virus Infection in Travelers Returning to United Kingdom from South Africa
Source: Emerg Infect Dis. 2019 Feb;25(2):367–9. doi: 10.3201/eid2502.172101 (PMC6346434; doi:10.3201/eid2502.172101)
Supplement: Appendix — Figure giving timeline of West Nile virus exposure risk, testing, and progress of illness in woman who returned to the United Kingdom from South Africa. [file 17-2101-Techapp-s1.pdf]

Article DOI: https://doi.org/10.3201/eid2502.172101

# West Nile Virus Infection in Travelers Returning to United Kingdom from South Africa

## Appendix

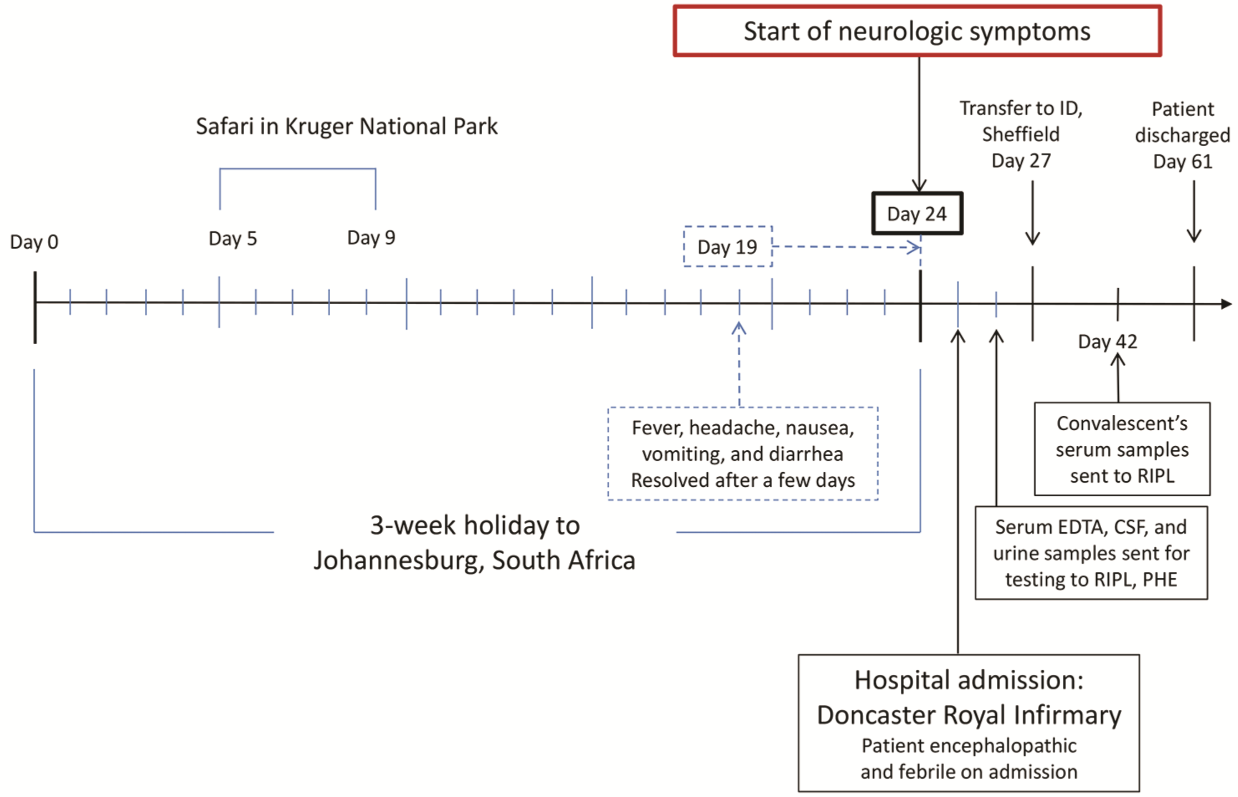


**Appendix Figure.** Timeline of West Nile virus exposure risk, testing, and progress of illness in woman who returned to the United Kingdom from South Africa. CSF, cerebrospinal fluid; EDTA, ethylenediaminetetraacetic acid; ID, Department of Infection and Tropical Medicine; PHE, Public Health England; RIPL, Rare and Imported Pathogens Laboratory.
